# σ^2^_*R*_, a reciprocal-space measure of the quality of macromolecular electron-density maps

**DOI:** 10.1107/S0907444999003583

**Published:** 1999-06-01

**Authors:** Thomas C. Terwilliger

**Affiliations:** a Structural Biology Group, Mail Stop M888, Los Alamos National Laboratory, Los Alamos, NM 87545, USA

**Keywords:** electron-density maps, phase quality, reciprocal space, roughness

## Abstract

It has previously been shown that the presence of distinct regions of solvent and protein in macromolecular crystals leads to a high value of the standard deviation of local r.m.s. electron density and that this can in turn be used as a reliable measure of the quality of macromolecular electron-density maps [Terwilliger & Berendzen (1999*a* ▶). *Acta Cryst.* D**55**, 501–505]. Here, it is demonstrated that a similar measure, , the variance of the local roughness of the electron density, can be calculated in reciprocal space. The formulation is suitable for rapid evaluation of macromolecular crystallographic phases, for phase improvement and for *ab initio* phasing procedures.

## Abstract 2

A reciprocal-space measure of the quality of macromolecular crystallographic phases based on the variance of the local roughness of the map is presented.

## 1. Introduction

A key step in the determination of macromolecular crystal structures, either by direct methods or by more traditional MAD or MIR approaches, is the evaluation of the quality of an electron-density map. In applying direct methods to macromolecular crystal structure determination, statistical relationships derived from characteristics of small-molecule structures (*e.g.* Sheldrick, 1990 ▶; Weeks *et al.*, 1995 ▶; Hauptman, 1997 ▶) are typically used to discriminate between possible phase sets. In the MAD or MIR approaches, the crystallographer typically manually examines an electron-density map and equates its interpretability with its quality. There would be considerable utility in having objective measures of the quality of electron-density maps which include as many features of macromolecular crystals as possible. Such measures could be used to choose between possible phase sets in *ab initio* methods and between possible heavy-atom solutions in the MIR and MAD methods. Additionally, if the measure of quality could be expressed in a simple reciprocal-space formulation, the measure could be used to improve phase quality or even to determine phases *ab initio*.

One measure of the quality of macromolecular electron-density maps which has been proposed is an automated analysis of the connectivity of electron-density maps (Baker *et al.*, 1993 ▶). This approach works well for evaluation of a map, but unfortunately it has proven difficult to use in phase improvement. We have recently demonstrated that an evaluation of the distinction between solvent and protein regions can be a very powerful criterion for scoring electron-density maps (Terwilliger & Berendzen, 1999*a* ▶,*b* ▶). Our approach is based on the well known observation that macromolecular crystals typically contain distinct regions of protein (where the local variation of electron density from point to point is very high) and solvent (where the electron density is essentially constant). This observation has been the basis of widely used solvent-flattening procedures (Wang, 1985 ▶; Xiang *et al.*, 1993 ▶; Podjarny *et al.*, 1987 ▶; Abrahams *et al.*, 1994 ▶; Zhang & Main, 1990 ▶).

We have used the difference between protein and solvent regions to generate an objective measure of the quality of a macromolecular electron-density map. Firstly, we calculated the local r.m.s. electron density near each grid point in the asymmetric unit, omitting the *F*_000_ term in the Fourier synthesis. In this way, the local r.m.s. density is very small in the solvent region but large in the protein region. We then determined the standard deviation of this local r.m.s. density over the entire asymmetric unit and use it as a figure of merit of the phasing. Maps which have a uniform distribution of local r.m.s. density have low values of the standard deviation; those with distinct protein and solvent regions have higher values. We have found this measure very useful in differentiating between heavy-atom solutions in the MIR and MAD approaches, as well as in identification of the hand of heavy-atom solutions when anomalous differences have been measured (Terwilliger & Berendzen, 1999*a* ▶).

Although it is difficult to express the standard deviation of local r.m.s. electron density in a reciprocal-space formulation, a very closely related characteristic, the variance of the local roughness, can be calculated readily. Here, we define this variance of the local roughness as the overall variance of the local variance of electron density in a map, and show how it can be calculated in reciprocal space. The expression we derive is suitable as a figure of merit for phase-quality evaluation, for phase improvement and for *ab initio* phasing methods.

## 2. Theory

In our previous work, we calculated the standard deviation of local r.m.s. electron density in a map. It was calculated using a grid with spacing approximately one-third of the resolution of the map in boxes five grid units on an edge, and the standard deviation of the local r.m.s. density was obtained from overlapping boxes throughout the asymmetric unit of the crystal (Terwilliger & Berendzen, 1999*a* ▶). We found that the precise size and overlap of the boxes had only small effects on the calculation. Here, we use a closely related but more generalizable approach, in which the overall variance of the local roughness of electron density is calculated. Instead of using overlapping boxes to determine the variation of local mean-square density from point to point in the cell, we use a windowing function to define the region over which the local variance (roughness) of electron density is calculated. Any windowing function could be used for this purpose, but a particularly convenient one is a Gaussian function.

The local roughness in a map [*r*(**x**)] can be represented by the weighted variance of electron density in a region defined by a windowing function centered at **x**: 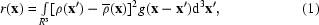
or equivalently 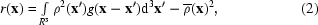
where 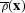 is the mean local electron density, given by 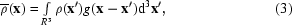
and *g*(**x**) is an arbitrary windowing function. If the windowing function is a three-dimensional Gaussian function with unit volume and a variance (for each of the components *x*, *y*, *z*) of σ^2^ then it can be expressed as 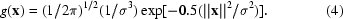
The variance (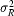) of this local roughness of electron density over the entire unit cell is then given by
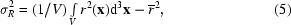
where 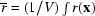 and *V* is the volume of the unit cell.

To calculate the variance of local roughness of the electron density, 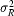, in reciprocal space, we use the facts that the first term on the right-hand side of (2) represents the convolution of ρ^2^(**x**) and *g*(**x**), and that 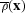 in (2) is in turn the convolution of ρ(**x**) and *g*(**x**). The electron density ρ(**x**), assumed to be a real function, and the squared electron density ρ^2^(**x**) can be expressed as (*cf*. Bracewell, 1986 ▶) 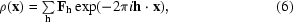
and 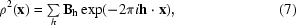
respectively, where **h** ≡ (*h* a*, *k* b*, *l* c*) and the reciprocal lattice vectors are a*, b* and c*. The coefficients **B_h_** can be calculated from the structure factors **F_h_** using the relation 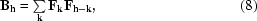
summing over all values of **k**. The Gaussian function *g*(**x**) can be readily expressed in Fourier space; it appears as the temperature factor in the Fourier transform of a Gaussian distribution of electron density about an atom, for example. An expression for a Gaussian centered at the origin with unit volume and a variance of ρ^2^ is 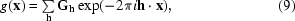
where 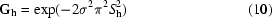
and *S* **_h_** is the magnitude of the scattering vector 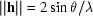.

As 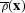 (3) is the convolution of ρ(**x**) and *g*(**x**), we can write 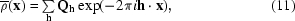
where the coefficients **Q_h_** are simply the original structure factors **F_h_** damped by the exponential factors **G_h_**, 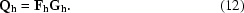
The second term on the right-hand side of (2) can now be expressed using (7) and (8) as 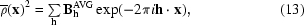
where the coefficients 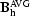 are based on the dampened structure factors **Q_k_** in (12),
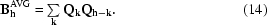
Next, as the first term on the right-hand side of (2) is the convolution of 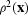 and *g*(**x**), we can write 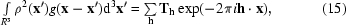
where the coefficients **T_h_** are given by 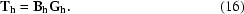
We can now express the local roughness of a map (1) in the form 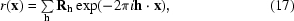
where the coefficients **R_h_** are given by 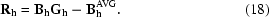
The desired variance 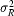 in (5) is composed of two parts, the mean value of *r* ^2^(**x**) and the square of the mean value of *r*(**x**) over the unit cell. The mean value of *r*(**x**) over the unit cell is simply the **h** = (0, 0, 0) term of its corresponding transform, **R**_000_. Similarly, the mean value of *r* ^2^(**x**) is given by the **h** = (0, 0, 0) term of its transform. Using Parseval’s theorem (*cf*. Bracewell, 1986 ▶), the mean value of *r* ^2^(**x**) can be expressed in the form 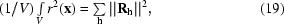
where the integral is taken over the unit-cell volume.

Finally, the variance of local roughness (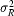) in (5) can be written as 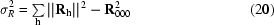
or more simply as
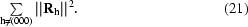

         

## 3. Discussion

(21) is a representation in reciprocal space of 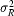, the variance of the local roughness of electron density in a Fourier synthesis. In the case of macromolecular crystals containing well defined regions of protein and solvent, this variance tends to be very high, as protein-containing areas of the unit cell are very rough and solvent-containing areas are very smooth (Terwilliger & Berendzen, 1999*a* ▶). Consequently, the value of this variance can be used as a measure of the relative qualities of various possible phase sets for a macromolecular structure.

The variance of local roughness, 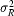, in (21) is given by the sum of squares of the coefficients **R_h_**, other than **R**_000_, in the Fourier synthesis for the local roughness, *r*(**x**). This is equivalent to noting that 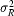 is simply the overall mean square value of the local roughness, after subtracting the overall average value of **R**_000_. The coefficients **R_h_** for the local roughness, given in (18), each contain two terms, **B_h_G_h_** and 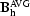. The first term, **B_h_G_h_**, consists of coefficients in the Fourier series expression (15) for the local mean-square electron density. The second term, 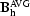, are coefficients in the Fourier series expression for the local mean electron density, squared. The difference corresponds to the local variance of the electron density, which we describe as local roughness.

An important feature of (21) is that only the low-order terms are large. This is a consequence of the presence of the exponential terms **G_h_** multiplying the **B_h_** terms in (18) and multiplying the **F_h_** terms in (12). Because of this, 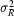 in (21) is, to a first approximation, the sum of the squares of the lowest-order terms in the Fourier series (7) describing 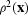. The magnitudes of these low-order terms describe how well defined the regions of the unit cell are which contain low and high values of 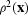. If the distribution of 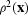 is relatively uniform in the unit cell, then the low-order terms in this Fourier series will be small. If the distribution is highly non-uniform then the low-order terms, and hence 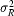, will be large.

(21) has several important properties which should be emphasized. The most significant is that the exponential term limits the range of **h** over which the terms in the summation are large to those with small 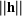. This means that evaluating 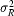 can be rapid. The calculation of each **B_h_** in (8) or 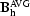 in (14) requires just one pass through all reflections. As only small values of **h** make a large contribution to 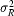, a relatively small number of passes through the reflections are necessary to calculate 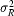. The potential rapidity of calculation of 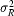 means that Monte Carlo methods or methods based on the genetic algorithm could potentially be used to optimize 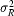 even in cases with large numbers of reflections. If a windowing function other than a Gaussian is used, or if the Gaussian function has a very narrow width, however, the number of terms needed to accurately evaluate 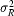 would not necessarily be small. In general, the calculation of 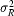 using the low-order terms in (21) corresponds to truncation of the spectrum of the windowing function at some resolution.

The second significant aspect of (21) is that the value of 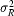 depends on the crystallographic phases in an easily calculable way. It is straightforward to differentiate (21) with respect to individual phases. This means that matrix methods can be used to adjust the phases to maximize 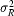. As reflections only interact significantly in (8) with other reflections which differ in **k** by a small number, such matrix methods would have to involve at most only a fraction of the elements in the matrix and possibly just diagonal elements. This kind of approach could be used to combine the maximization of 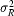 with that of other direct-methods figures of merit to improve the ability of direct-methods to solve macromolecular structures.

**Figure 1:**
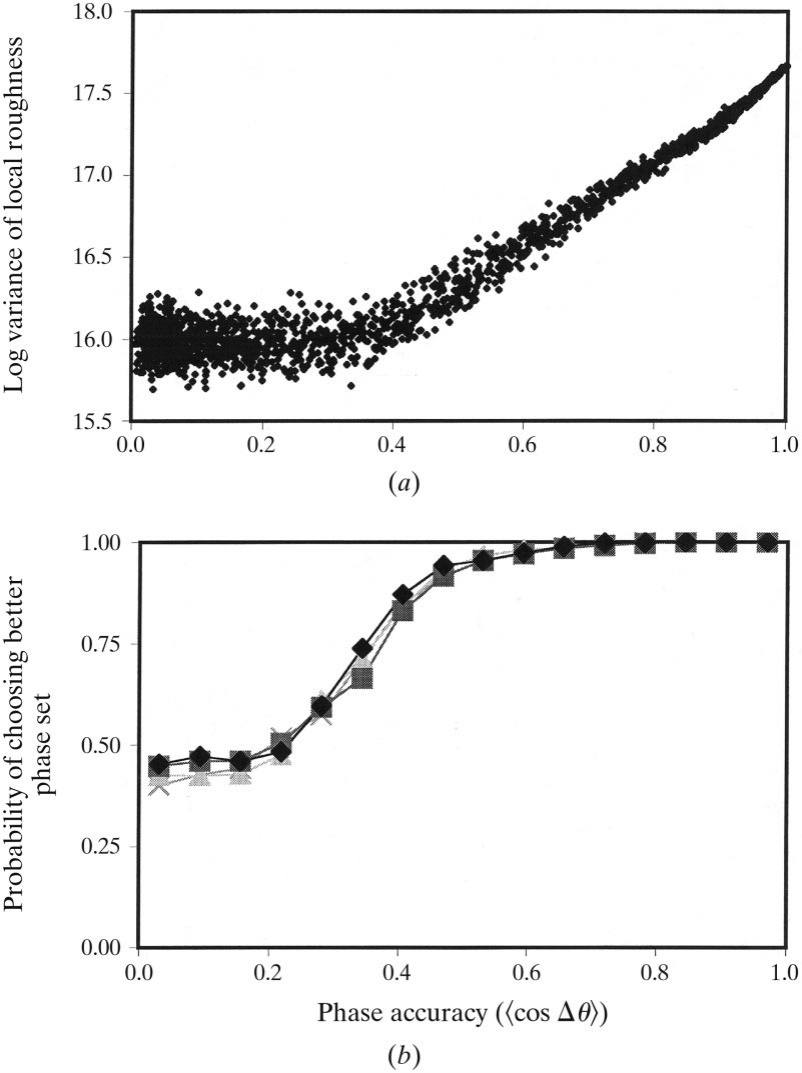
Calculation of variance of local roughness using (21). (*a*) The logarithm of 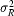 is plotted for 2000 model phase sets, as described in the text. The abscissa is 〈cos(Δθ)〉, the mean value of the effective figure of merit of the phase set. (*b*) The probability of choosing the better of two phase sets which differ in quality by 0.1 units of 〈cos(Δθ)〉 is plotted for model data obtained as in (*a*), using the 364 lowest order terms (diamonds), 249 lowest order terms (triangles), 145 lowest order terms (squares) or 58 lowest order terms (crosses), as described in the text.

As (21) is essentially a reciprocal-space formulation of the real-space measure of map quality which we have already examined in detail (Terwilliger & Berendzen, 1999*a* ▶), most of the properties of the two formulations will be very similar. In Fig. 1 ▶, we present a set of model calculations using (21) to evaluate electron-density maps in reciprocal space. 6200 model data from 20 to 3.0 Å were generated based on coordinates from a dehalogenase enzyme from *Rhodococcus* species ATCC 55388 (American Type Culture Collection, 1992 ▶) determined recently in our laboratory. The protein contains 316 amino-acid residues and crystallizes in space group *P*2_1_2_1_2 with unit-cell dimensions *a* = 94, *b* = 80, *c* = 43 Å and one molecule in the asymmetric unit (J. Newman, personal communication). Fig. 1 ▶(*a*) shows results for a total of 2000 phase sets generated from the model data, with phase errors ranging from 0–150°. These model data sets were analyzed using (21) with a value of σ = 6 Å and including all 364 terms for which the exponential term G(**h**) in (10) has a value of 0.0001 or larger. The logarithm of the variance in local roughness, log(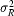), is plotted in Fig. 1 ▶(*a*) as a function of the cosine of the mean phase error in the data set. For phase sets with 〈cos(Δθ)〉 of ∼0.3 or greater, the logarithm of the variance in local roughness is quite closely related to the phase accuracy. For phase sets with lower 〈cos(Δθ)〉, there is only a small correlation.

Fig. 1 ▶(*b*) shows the practical implications of the data in Fig. 1 ▶(*a*) and also illustrates that only low-order terms in (21) are necessary for calculating 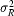. In Fig. 1 ▶(*b*), the data in Fig. 1 ▶(*a*) are analyzed to estimate the probability that a correct choice of the better of two phase sets can be determined from the logarithm of the variance of local roughness. Fig. 1 ▶(*b*) shows analyses of four groups of 2000 phase sets each. In each of the four analyses, a different minimum value of the exponential term G(**h**) was used, ranging from 0.0001 to 0.1. To obtain Fig. 1 ▶(*b*), the data in Fig. 1 ▶(*a*) were grouped into pairs of sets differing by 0.1 ± 0.05 units in 〈cos(Δθ)〉. Each member of each set was compared with each member of the paired set, and the fraction of times that the member with the higher value of log(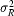) also had the higher value of 〈cos(Δθ)〉 was plotted.

## 

Fig. 1 ▶(*b*) shows that, as expected in phase sets with very low phase accuracy (〈cos(Δθ)〉 < 0.25), the value of log(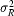) leads to only a 50% chance of choosing the better of two phase sets which differ in accuracy. For phase sets with values of 〈cos(Δθ)〉 from 0.25 to 0.4, however, the probability of choosing the better of two phase sets differing by this amount increases from 0.6 to 0.9. The 58 lowest order terms in the series in (21) give almost the same likelihood of making a correct choice as the 364 lowest order terms. This means that high-order terms can be ignored without a substantial effect.

## 4. Conclusions

The reciprocal-space formulation presented here has major advantages compared with the real-space calculations carried out previously (Terwilliger & Berendzen, 1999*a* ▶). These are that the variance 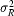 can be calculated without a Fourier transform and that potentially phases can be adjusted to maximize the variance. The rapid calculation of variance means that it can be used as a measure of the quality of phases in many different trials, and the potential for maximization of the variance means that it can be used in phase improvement and possibly even *ab initio* phasing algorithms. The most powerful means for phase improvement for macromolecules without non-crystallographic symmetry is at present solvent flattening (Wang, 1985 ▶; Xiang *et al.*, 1993 ▶; Podjarny *et al.*, 1987 ▶; Abrahams *et al.*, 1994 ▶; Zhang & Main, 1990 ▶). Carrying out this type of procedure requires that the electron-density map be of sufficiently high quality that an envelope defining the boundary between protein and solvent can be reliably calculated. (21) provides a means for improving phases even before the boundary is clearly defined. Maximizing 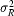 will maximize the distinction between protein and solvent regions without requiring a knowledge of where each are located. Consequently, (21) may be useful in cases where solvent flattening is not effective, as well as providing a complementary approach in cases where phases are good to begin with.

There are several aspects of the reciprocal-space formulation which remain to be optimized. One is the choice of the windowing function. We have chosen a Gaussian function, but the derivation we carried out is independent of the windowing function and any function could have been used. A Gaussian is particularly convenient because it results in strongly damped coefficients that become very small for all but small values of 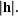 Other windowing functions, however, might yield better measures of the quality of the electron-density map, and a survey of other functions might improve the algorithm. Another possibility might be to construct a histogram of values of 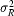 from many solved protein structures which could in turn be used to construct a data-likelihood model for estimation of phase errors. Such an approach could be considerably more powerful than the one described here because it would give probability information which could be combined in a Bayesian approach with other sources of phase information.
